# Whole-lesion apparent diffusion coefficient (ADC) histogram as a quantitative biomarker to preoperatively differentiate stage IA endometrial carcinoma from benign endometrial lesions

**DOI:** 10.1186/s12880-022-00864-9

**Published:** 2022-08-08

**Authors:** Jieying Zhang, Xiaoduo Yu, Xiaomiao Zhang, Shuang Chen, Yan Song, Lizhi Xie, Yan Chen, Han Ouyang

**Affiliations:** 1 Department of Diagnostic Radiology, National Cancer Center/National Clinical Research Center for Cancer/Cancer Hospital, Chinese Academy of Medical Sciences and Peking Union Medical College, Beijing, 100021 China; 2 Department of Pathology, National Cancer Center/National Clinical Research Center for Cancer/Cancer Hospital, Chinese Academy of Medical Sciences and Peking Union Medical College, Beijing, 100021 China; 3 MR Research China, GE Healthcare, Beijing, 100176 China

**Keywords:** Endometrial neoplasms, Apparent diffusion coefficient, Diffusion-weighted MRI, Histogram analysis

## Abstract

**Background:**

To assess the value of whole-lesion apparent diffusion coefficient (ADC) histogram analysis in differentiating stage IA endometrial carcinoma (EC) from benign endometrial lesions (BELs) and characterizing histopathologic features of stage IA EC preoperatively.

**Methods:**

One hundred and six BEL and 126 stage IA EC patients were retrospectively enrolled. Eighteen volumetric histogram parameters were extracted from the ADC map of each lesion. The Mann–Whitney U or Student’s t-test was used to compare the differences between the two groups. Models based on clinical parameters and histogram features were established using multivariate logistic regression. Receiver operating characteristic (ROC) analysis and calibration curves were used to assess the models.

**Results:**

Stage IA EC showed lower ADC_10th_, ADC_90th_, ADC_min_, ADC_max_, ADC_mean_, ADC_median_, interquartile range, mean absolute deviation, robust mean absolute deviation (rMAD), root mean squared, energy, total energy, entropy, variance, and higher skewness, kurtosis and uniformity than BELs (all *p* < 0.05). ADC_median_ yielded the highest area under the ROC curve (AUC) of 0.928 (95% confidence interval [CI] 0.895–0.960; cut-off value = 1.161 × 10^−3^ mm^2^/s) for differentiating stage IA EC from BELs. Moreover, multivariate analysis demonstrated that ADC-score (ADC_10th_ + skewness + rMAD + total energy) was the only significant independent predictor (OR = 2.641, 95% CI 2.045–3.411; *p* < 0.001) for stage IA EC when considering clinical parameters. This ADC histogram model (ADC-score) achieved an AUC of 0.941 and a bias-corrected AUC of 0.937 after bootstrap resampling. The model performed well for both premenopausal (accuracy = 0.871) and postmenopausal (accuracy = 0.905) patients. Besides, ADC_min_ and ADC_10th_ were significantly lower in Grade 3 than in Grade 1/2 stage IA EC (p = 0.022 and 0.047). At the same time, no correlation was found between ADC histogram parameters and the expression of Ki-67 in stage IA EC (all p > 0.05).

**Conclusions:**

Whole-lesion ADC histogram analysis could serve as an imaging biomarker for differentiating stage IA EC from BELs and assisting in tumor grading of stage IA EC, thus facilitating personalized clinical management for premenopausal and postmenopausal patients.

**Supplementary Information:**

The online version contains supplementary material available at 10.1186/s12880-022-00864-9.

## Background

A global increase in the prevalence of endometrial pathologies parallels escalating levels of obesity, progressive aging of the population, and increasing trends in delaying childbearing [1, 2]. In clinical practice, patients are diagnosed with suspected endometrial lesions due to abnormal uterine bleeding, infertility, or even an abnormal appearance of the endometrium as an incidental finding on imaging performed for other indications [3–5]. It is crucial to make an accurate preoperative diagnosis of endometrial lesions for radiologists and gynecologists, thus avoiding unnecessary surgical procedures and protecting the patients’ fertility.

Endometrial sampling biopsy with curettage or hysteroscopy serves as the primary diagnostic approach. Still, this method is invasive and not always possible (e.g., for patients with cervical stenosis or those unable to tolerate the procedure). Transvaginal ultrasound is an alternate cost-efficient examination and usually the first choice, but it has a relatively low specificity and depends largely on the operators [6, 7]. Magnetic resonance imaging (MRI) is recommended for cases with inconclusive sonographic findings. Nevertheless, pre-surgical evaluation of uterine cavity abnormalities by conventional MRI remains challenging [8]. This difficulty can be attributed to the variable and potentially overlapping imaging features of a large spectrum of benign and malignant endometrial lesions [9].

Apparent diffusion coefficient (ADC) values obtained from diffusion-weighted imaging (DWI) have shown potency in characterizing endometrial pathologies as benign or malignant [10–13]. The ADC measures the random motion of water molecules and decreases with increasing tumor cellularity, as seen in malignant lesions [14]. However, drawing a region of interest on a representative section of tumors may impact the ADC values and interobserver variability [15, 16].

The whole-lesion histogram-based ADC analysis offers a more comprehensive assessment of a given abnormality than traditional ADC measures and provides an option for quantifying the overall heterogeneity of the tumor. Recently, ADC histogram analysis has been increasingly applied in genitourinary imaging [17–19] to differentiate the histological types, predict tumor grade, and assess treatment response. A previous study has demonstrated that ADC histogram metrics may help radiologists differentiate benign from malignant endometrial lesions in premenopausal patients [20] while limited by fairly small sample size (n = 54). To the best of our knowledge, no studies have reported on ADC histogram analysis for differentiating benign endometrial lesions (BELs) from the International Federation of Gynecology and Obstetrics (FIGO) stage IA endometrial carcinoma (EC, the tumor is limited to the uterine corpus without or with less than 50% myometrial invasion). Moreover, several studies have shown that ADC histogram parameters may reflect different histopathological features and assist in evaluating tumor proliferation. For instance, ADC histogram parameters have recently been reported to be associated with the expression of Ki-67, epidermal-growth factor (EGFR), and histone 3 in uterine cervical cancer [21, 22], and also the expression of p53 in epithelial ovarian cancer [23].

Therefore, we aimed to evaluate the role of whole-lesion ADC histogram analysis in differentiating stage IA EC from BELs in premenopausal and postmenopausal patients and characterizing histopathologic features of stage IA EC preoperatively.

## Methods

### Study population

This retrospective study was approved by our institutional review board, and the requirement for written informed consent was waived. After reviewing the medical records of our hospital between January 2011 and December 2019, 232 patients diagnosed with BELs and EC were selected. All lesions were pathologically confirmed after hysterectomy or hysteroscopic resection. The benign pathologies included endometrial polyp, endometrial hyperplasia without atypia, and atypical endometrial hyperplasia. All of the ECs were in FIGO 2018 stage IA.

**Fig. 1:**
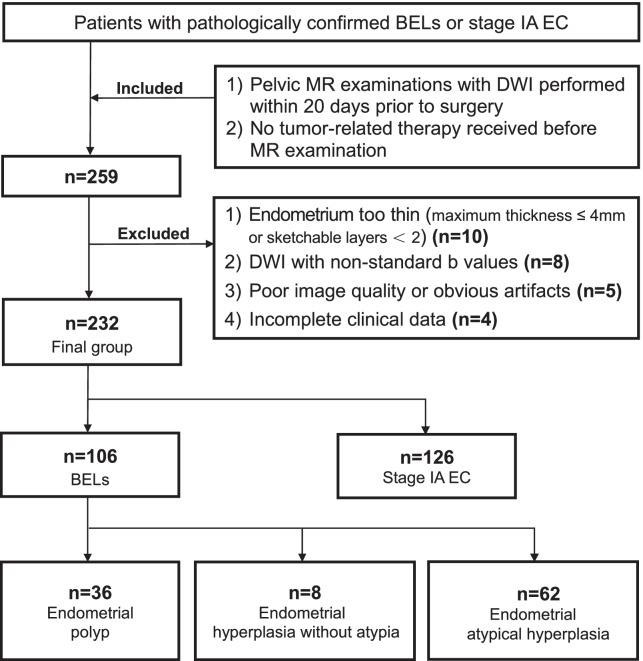
The flowchart of patient enrollment

Inclusion criteria were: (1) pelvis MR imaging with DWI performed within 20 days before surgery; (2) no tumor-related therapy received before MR examination. Exclusion criteria were the following: (1) endometrium too thin (maximum thickness was less than 5 mm on sagittal T2-weighted images or sketchable layers were less than two) to be accurately measured; (2) DWI with non-standard b values (other than 0, 800 s/mm^2^); (3) poor image quality or noticeable artifacts; (4) incomplete clinical data. The flowchart of patient enrollment is shown in Fig. 1.

### MRI protocol

All pelvis MR examinations were performed using 3.0T scanners (Signa HDxt and Discovery MR 750, GE Medical System) equipped with an eight-element phased coil with patients in the supine position. Patients with no contraindications received 10 mg raceanisodamine hydrochloride injection intramuscularly before image acquisition to reduce the bowel motion artifacts. DWI was obtained in the axial plane using a single-shot echo-planar imaging technique before the injection of the contrast agent. Diffusion gradients were applied in three orthogonal directions with b values of 0 and 800 s/mm^2^. More detailed sequence scanning parameters are shown in Table 1.

**Table 1:** MR imaging protocol

	Axial T1WI	Axial T2WI	Sagittal T2WI	Axial oblique T2WI	Axial DWI*	Axial T1WI postcontrast
**GE signa excite HD 3.0T**						
Technique	FSE	FS FSE	FSE	FSE	SS-EPI	LAVA
TR (ms)/TE (ms)	620/8.2	5900/121	4920/139.1	4900/131.5	4400/64.3	4.1/1.8
Field of view (cm)	38.0	34.0	30.0	22.0	34.0	35.0
Matrix (phase × frequency)	320 × 224	320 × 256	320 × 256	320 × 256	256 × 256	350 × 350
Slice thickness (mm)	5	5	4	3	5	1
Slice gap	1	1	0.4	0	1	0
b value(s/mm^2^)	–	–	–	–	0, 800	–
**GE Discovery HD750 3.0T**						
Technique	LAVA-Flex	FS FSE	FSE	FSE	SS-EPI	LAVA
TR (ms)/TE (ms)	4.2/1.3	5100/106.6	5620/135.6	5500/102.0	5500/62.7	7.9/4.1
Field of view (cm)	34.0	34.0	30.0	22.0	34.0	35.0
Matrix (phase × frequency)	320 × 224	320 × 256	320 × 256	320 × 256	128 × 128	350 × 350
Slice thickness (mm)	3	5	4	3	5	1
Slice gap	0	1	0.4	0	1	0
b value (s/mm^2^)	–	–	–	–	0, 800	–

T1WI, T1-weighted imaging; T2WI, T2-weighted imaging; FS, fat suppression; FSE, fast-recovery fast spin echo; DWI, diffusion-weighted imaging; SS-EPI, single-shot echo-planar imaging; LAVA-Flex, liver acquisition with volume acceleration-flexible; GRE, gradient echo; TR, repetition time; TE, echo time

*ADC maps were calculated voxel by voxel with the monoexponential model using formula: ${\text{ADC}} = {\text{ln}}\left( {S0/{\text{S}}800} \right)/\left( {b800 - b0} \right)$ where *S800* and *S0* are the signal intensities with and without a diffusion gradient, respectively

### Imaging analysis

ADC maps were manually generated from DWI on the post-processing workstation (Advantage Workstation 4.6; GE Medical System). Two radiologists (J.Z. and X.Y., with 6- and 18-years’ experience in gynecologic MR imaging, as reader 1 and 2) retrospectively reviewed all images independently while blinded to the clinical and pathological information.

The ITK-SNAP software (version 3.8.0, www.itksnap.org) was used in this study. The volume of interest (VOI) covering the whole tumor was manually drawn along the boundary of the tumor or entire endometrium (if without visible tumor) on all slices of DWI images (b = 800 s/mm^2^) by reader 1. T1-weighted images, T2-weighted images, and dynamic contrast-enhanced images were used as references to avoid the necrotic, cystic, hemorrhagic areas and adjacent normal tissues being included in the VOIs. Then, the VOIs were automatically copied to ADC maps. After a one-month interval, we repeated the drawing by reader 1 and 2 independently. Inter- and intra-observer agreements of ADC histogram metrics were determined by calculating the intraclass correlation coefficients (ICC). Cases with apparent inconsistent VOIs between reader 1 and 2 were reassessed by another radiologist (H.O., with 30-years’ experience in gynecologic imaging) to ensure high-quality final segmentation results.

The ADC histogram analysis was performed with the open-source PyRadiomics software [24] to obtain the volume of tumors and 18 first-order parameters, including 10th percentile ADC (ADC_10th_), 90th percentile ADC (ADC_90th_), ADC_min_, ADC_max_, ADC_mean_, ADC_median_, interquartile range (IQR), range, mean absolute deviation (MAD), robust mean absolute deviation (rMAD), root mean squared (RMS), energy, total energy, entropy, skewness, kurtosis, variance, and uniformity. Image normalization was applied on the ADC map before parameter extraction using the PyRadiomics normalization method by centering it at the mean with standard deviation based on all gray values in the image (not just those inside the segmentation).

### Histopathologic analysis

A pathologist (Y.S.) with 20 years’ experience in gynecologic pathology reviewed the postoperative pathological data, including tumor classification, grading, and Ki-67 testing, while blinded to the clinical and image data. The EC tumor grade was established first (Grade 1 = well differentiated; Grade 2 = moderately differentiated; Grade 3 = poorly differentiated). According to the literature [25], the following histological subtypes of EC were classified as Grade 3: serous carcinoma, clear cell carcinoma, mixed carcinoma, and carcinosarcomas. Then, the tumors were divided into two groups: high-grade (Grade 3) and low-grade (Grade 1 and 2). The Ki-67 labeling index was estimated by counting positively stained nuclei of tumor cells number in all pictures per lesion. A cut-off value of 30% was used to divide Ki-67 expression into the low-proliferation group (< 30%) and high-proliferation group (≥ 30%) [26, 27].

### Statistical analysis

Categorical variables were analyzed using the chi-square or Fisher’s exact test when the expected value in any cell was less than five. Continuous variables were analyzed using a t-test or Mann–Whitney U test after checking for normality using the Kolmogorov–Smirnov test. The receiver operating characteristic (ROC) curves were performed for all significant variables to assess the differential diagnostic efficiency of these features. Based on ROC analysis, the optimal cut-off value was determined using the maximum Youden index (i.e., sensitivity + specificity − 1).

Before the feature selection process, we applied Z score normalization (standardization) to ensure that the histogram parameters were measured on the same scale. The variables with a *p* < 0.1 on univariate analysis were further analyzed using multivariate analysis. After that, the multivariate logistic regression analysis with a forward stepwise selection procedure was used to select and construct different diagnostic models. The diagnostic performance of the model was assessed using the ROC curves with the corresponding AUC, sensitivity, specificity, and accuracy. Calibration curves and the Hosmer–Lemeshow test were used to evaluate the goodness-of-fit of models. DeLong’s test was used to compare the AUC of each model. Model internal validations were performed using the enhanced bootstrap resampling method (n = 1000), which obtained the estimates of optimism in the regression models to provide a bias-corrected AUC value. Spearman's rank correlation coefficient was used to calculate the correlation between ADC histogram parameters and the Ki-67 labeling index. Statistical analyses were performed using R software (version 4.0.3; http://www.Rproject.org). A *p* < 0.05 was considered statistically significant.

## Results

### Patient characteristics

**Fig. 2:**
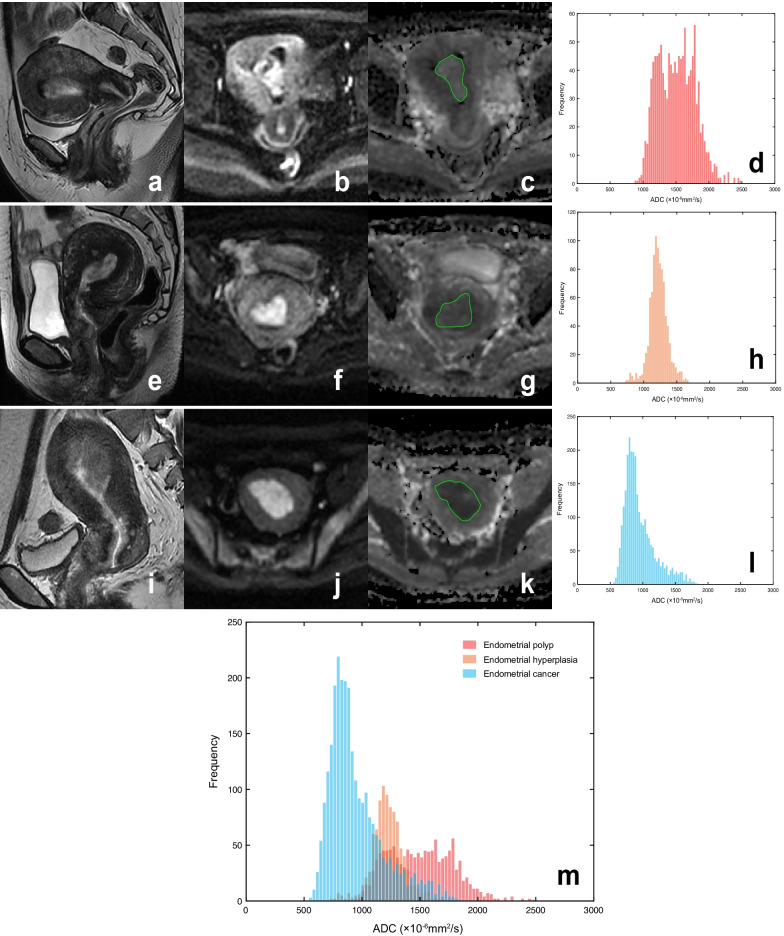
Findings of three patients with histopathological-proven endometrial polyp (**a–d**), atypical endometrial hyperplasia (**e**–**h**), and stage IA EC (**i**–**l**). Sagittal T2WI (**a**, **e**, **i**), axial DWI (b = 800 s/mm^2^; **b**, **f**, **j**), axial ADC maps (**c**, **g**, **k**), and ADC histogram (**d**, **h**, **l**). All three lesions showed moderate hyperintensity on T2WI and hyperintensity on DWI. The BELs (endometrial polyp and hyperplasia) showed slight hyperintensity or isointensity, while stage IA EC showed hypointensity on the ADC maps. **m** The ADC histograms reflect the differences in the frequency of voxels distribution between benign and malignant endometrial tumors

A total of 232 patients were enrolled in our study, including 106 BEL patients (age range, 34–77 years; median age, 49 years) and 126 stage IA EC patients (age range, 28–77 years; median age, 53 years). Tables 2 and 3 show patients’ clinical and histopathological characteristics, respectively. Representative cases of BELs and stage IA EC are presented in Fig. 2.

**Table 2:** Summary of patients' clinical characteristics

Characteristics	BELs (n = 106)	EC (n = 126)	*p* value
Age at diagnosis, years^†^			< 0.001*
≤ 50	65 (61.3)	42 (33.3)	
51–64	34 (32.1)	77 (61.1)	
≥ 65	7 (6.6)	7 (5.6)	
BMI, kg/m^2†^			0.405
≤ 24.9	48 (45.3)	49 (38.9)	
25–29.9	42 (39.6)	50 (39.7)	
≥ 30	16 (15.1)	27 (21.4)	
Menopausal status^†^			0.065
Premenopausal	60 (56.6)	56 (44.4)	
Postmenopausal	46 (43.4)	70 (55.6)	
Nulliparity^†^			0.092
No	103 (97.2)	116 (92.1)	
Yes	3 (2.8)	10 (7.9)	
Diabetes^†^			0.698
No	99 (93.4)	116 (92.1)	
Yes	7 (6.6)	10 (7.9)	
PCOS^†^			0.358
No	106 (100)	125 (99.2)	
Yes	0 (0)	1 (0.8)	
Long-term tamoxifen therapy^†^			0.001*
No	91 (85.8)	123 (97.6)	
Yes	15 (14.2)	3 (2.4)	
CA125(+)^†^	11 (10.4)	14 (11.1)	0.858
CA199(+)^†^	3 (2.8)	11 (8.7)	0.06

BELs, benign endometrial lesions; EC, endometrial cancer; BMI, body mass index; PCOS, polycystic ovary syndrome; CA125, cancer antigen 125; CA199, cancer antigen 199

^†^Data in parentheses are percentages. *p < 0.05

**Table 3:** Histopathological features of stage IA EC

Groups	N (%)
Subtype (n = 126)	
Endometrioid carcinoma	111 (88.1)
Mucinous carcinoma	5 (4.0)
Serous carcinoma	2 (1.6)
Clear cell carcinoma	1 (0.8)
Mixed carcinoma	4 (3.2)
Carcinosarcoma	3 (2.4)
Grade (n = 126)	
Grade 1	32 (25.4)
Grade 2	66 (52.4)
Grade 3	28 (22.2)
Low grade (Grade 1/2)	98 (77.8)
High grade (Grade 3)	28 (22.2)
Ki-67 labeling index (n = 80)	
Low expression (< 30%)	29 (36.3)
High expression (≥ 30%)	51 (63.7)

### Reliability of ADC histogram analysis

All measurements of whole-lesion histogram analysis showed excellent intraobserver and interobserver reliability (ICC = 0.955–0.998 and ICC = 0.926–0.997, respectively; Table 4).

**Table 4:** Comparison of ADC histogram parameters between stage IA EC and BELs

Histogram parameters	BELs (n = 106) ^*^	ECs (n = 126) ^*^	*p* value	Intra-observer ICC (95%CI)	Inter-observer ICC (95%CI)
Volume (× 10^3^ mm^3^)	6.076 (2.511, 10.477)	4.803 (2.533, 10.283)	0.581	0.985 (0.976–0.991)	0.972 (0.956–0.987)
ADC_10th_ (× 10^−3^ mm^2^/s)	1.129 (1.047, 1.236)	0.815 (0.722, 0.884)	< 0.001	0.997 (0.996–0.998)	0.996 (0.995–0.998)
ADC_90th_ (× 10^−3^ mm^2^/s)	1.521 (1.401,1.775)	1.189 (1.081, 1.312)	< 0.001	0.994 (0.993–0.996)	0.992 (0.990–0.995)
ADC_min_ (× 10^−3^ mm^2^/s)	0.945 ± 0.164	0.697 ± 0.149	< 0.001	0.965 (0.929–0.983)	0.953 (0.902–0.978)
ADC_max_ (× 10^−3^ mm^2^/s)	1.788 (1.549, 2.089)	1.511 (1.349, 1.704)	< 0.001	0.982 (0.964–0.992)	0.978 (0.955–0.990)
ADC_mean_ (× 10^−3^ mm^2^/s)	1.314 (1.219, 1.492)	0.989 (0.885, 1.083)	< 0.001	0.998 (0.996–0.998)	0.997 (0.995–0.998)
ADC_median_ (× 10^−3^ mm^2^/s)	1.315 (1.206, 1.458)	0.970 (0.872, 1.057)	< 0.001	0.998 (0.996–0.999)	0.996 (0.995–0.998)
IQR (× 10^−3^ mm^2^/s)	0.212 (0.167, 0.277)	0.191 (0.155, 0.237)	0.014	0.974 (0.946–0.988)	0.965 (0.927–0.983)
Range (× 10^−3^ mm^2^/s)	0.854 (0.655, 1.115)	0.802 (0.629, 1.027)	0.164	0.974 (0.946–0.987)	0.976 (0.950–0.989)
MAD (× 10^–3^)	0.128 (0.107, 0.166)	0.112 (0.098, 0.144)	0.013	0.992 (0.982–0.996)	0.988 (0.976–0.994)
rMAD (× 10^–3^)	0.088 (0.074, 0.115)	0.079 (0.064, 0.099)	0.007	0.990 (0.978–0.995)	0.985 (0.968–0.993)
RMS (× 10^–3^)	1.324 (1.229, 1.507)	1.003 (0.896, 1.100)	< 0.001	0.998 (0.996–0.998)	0.997 (0.996–0.998)
Energy (× 10^–3^)	0.781 (0.364, 1.599)	0.354 (0.195, 0.720)	< 0.001	0.997 (0.992–0.998)	0.996 (0.994–0.999)
Total energy (× 10^–3^)	11.134(4.152, 19.947)	4.926 (2.454, 10.009)	< 0.001	0.997 (0.994–0.999)	0.997 (0.994–0.999)
Entropy (× 10^–6^)	4.225 ± 0.582	4.038 ± 0.425	0.007	0.994 (0.987–0.997)	0.965 (0.927–0.983)
Skewness (× 10^–6^)	0.215 ± 0.522	0.744 ± 0.603	< 0.001	0.980 (0.959–0.990)	0.970 (0.935–0.986)
Kurtosis (× 10^–6^)	2.960 (2.438, 3.345)	3.377 (2.743, 4.451)	< 0.001	0.955 (0.906–0.979)	0.926 (0.891–0.954)
Variance (× 10^–6^)	0.027(0.017, 0.042)	0.022 (0.015, 0.034)	0.030	0.989 (0.977–0.995)	0.987 (0.972–0.994)
Uniformity (× 10^–6^)	0.065(0.049, 0.078)	0.072 (0.059, 0.087)	0.002	0.984 (0.967–0.992)	0.982 (0.961–0.991)

IQR, interquartile range; MAD, mean absolute deviation; rMAD, robust mean absolute deviation; RMS, root mean squared; ICC, intraclass correlation coefficients

^*^Data are mean ± standard deviation (normal distribution) or median and interquartile range (non-normal distribution), evaluated using the Kolmogorov–Smirnov test

### Comparison of ADC histogram parameters between BELs and stage IA EC

**Fig. 3:**
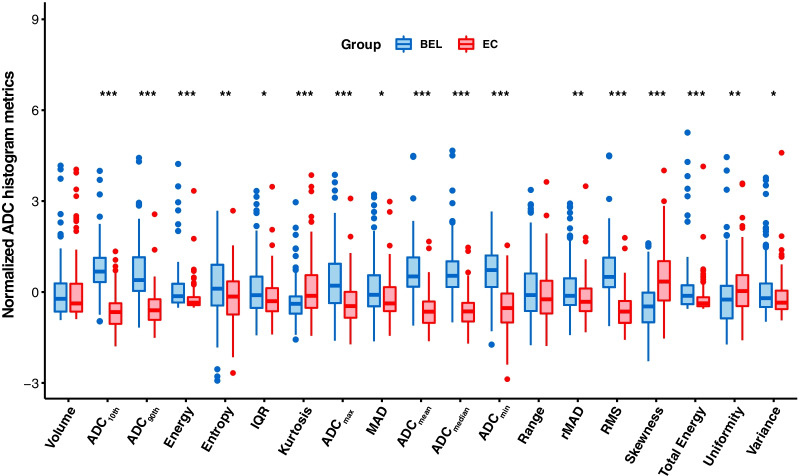
Boxplots graphically depict the quartiles and distributions of normalized volumetric ADC histogram parameters between BELs and stage IA EC. The colors are grouped based on histopathological results: BEL in red and EC in blue. *p < 0.05, **p < 0.01, ***p < 0.001. IQR, interquartile range; MAD, mean absolute deviation; rMAD, robust mean absolute deviation; RMS, root mean squared

**Fig. 4:**
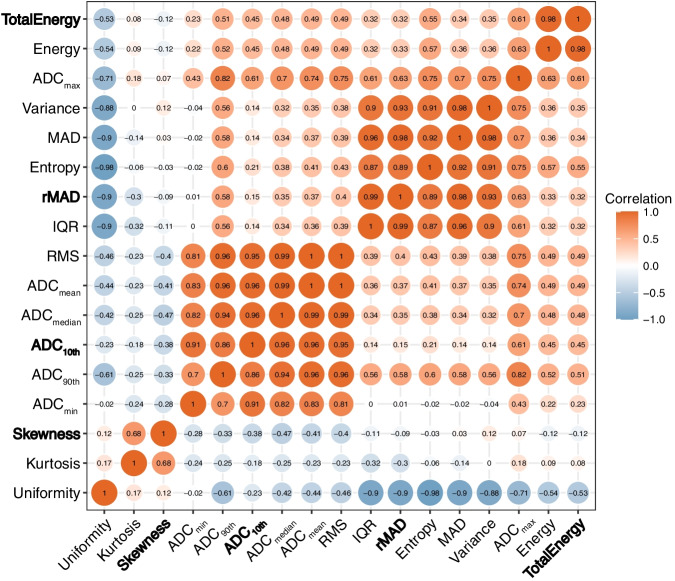
Spearman correlation coefficients of the significant ADC histogram parameters. The text in bold showed the parameters included in the ADC histogram model. IQR, interquartile range; MAD, mean absolute deviation; rMAD, robust mean absolute deviation; RMS, root mean squared

The results and distribution of ADC histogram parameters between stage IA EC and BELs are shown in Table 4 and Fig. 3. The ADC values of the stage IA EC, including ADC_10th_, ADC_90th_, ADC_min_, ADC_max_, ADC_mean_, ADC_median_, and IQR, were all significantly lower than those of BELs (all *p* < 0.05). The MAD, rMAD, RMS, energy, total energy, entropy, and variance of the stage IA EC were also significantly lower than those of BELs, and the skewness, kurtosis, and uniformity were higher (all *p* < 0.05). There were no significant differences in the tumor volume and range between stage IA EC and BELs (all *p* > 0.05). Spearman correlation coefficients of the significant ADC histogram parameters are shown in Fig. 4.

### Diagnostic efficacy of ADC histogram parameters in differentiating BELs and stage IA EC

The results of ROC curve analysis are summarized in Table 5. For the discrimination of stage IA EC from BELs, ADC_median_ generated the highest AUC (AUC = 0.928; 95% CI 0.895–0.960; cut-off value = 1.161 × 10^−3^ mm^2^/s; sensitivity = 88.9%; specificity = 83.0%), followed by ADC_mean_, RMS, and ADC_10th_ (AUC = 0.926, 0.925, and 0.920, respectively).

**Table 5:** Diagnostic performance of ADC histogram parameters in differentiating between stage IA EC and BELs

Histogram features	Cut-off	AUC (95%CI)	Sensitivity (%)	Specificity (%)	Accuracy (%)
ADC_10th_ (× 10^−3^ mm^2^/s)	0.973	0.920 (0.885–0.956)	86.5	85.8	86.2
ADC_90th_ (× 10^−3^ mm^2^/s)	1.401	0.891 (0.849–0.933)	88.9	75.5	82.8
ADC_min_ (× 10^−3^ mm^2^/s)	0.829	0.876 (0.830–0.922)	84.9	80.2	82.8
ADC_max_ (× 10^−3^ mm^2^/s)	1.627	0.747 (0.684–0.810)	68.3	68.9	68.5
ADC_mean_ (× 10^−3^ mm^2^/s)	1.147	0.926 (0.893–0.960)	86.5	87.8	87.1
ADC_median_ (× 10^−3^ mm^2^/s)	1.161	0.928 (0.895–0.960)	88.9	83	86.2
IQR (× 10^−3^ mm^2^/s)	0.193	0.594 (0.520–0.668)	52.4	64.2	57.8
MAD (× 10^–3^)	0.111	0.595 (0.521–0.669)	48.4	70.8	58.6
rMAD (× 10^–3^)	0.08	0.603 (0.529–0.676)	54	66	59.5
RMS (× 10^–3^)	1.157	0.925 (0.890–0.959)	85.7	86.8	86.2
Energy (× 10^–3^)	0.366	0.673 (0.603–0.743)	76.2	55.7	66.8
Total Energy (× 10^–3^)	9.91	0.663 (0.592–0.734)	74.6	57.5	66.8
Entropy (× 10^–6^)	4.082	0.609 (0.534–0.683)	55.6	65.1	59.9
Skewness (× 10^–6^)	0.566	0.748 (0.685–0.811)	63.5	80.2	71.1
Kurtosis (× 10^–6^)	3.357	0.649 (0.579–0.720)	52.4	77.4	63.8
Variance (× 10^–6^)	0.063	0.583 (0.508–0.657)	95.2	20.8	61.2
Uniformity (× 10^–6^)	0.072	0.620 (0.547–0.694)	52.4	70.8	60.8

IQR, interquartile range; MAD, mean absolute deviation; rMAD, robust mean absolute deviation; RMS, root mean squared

### Multivariate logistic regression models based on clinical and ADC histogram parameters

#### Clinical model

In the clinical model, multivariate regression analysis showed that age (51–64 years) (odds ratio [OR] = 4.106, 95% confidence interval [CI] 2.269–7.429; *p* < 0.001), nulliparity (OR = 1.433, 95% CI 1.066–16.472; *p* = 0.040), and long-term tamoxifen therapy (OR = 0.140, 95% CI 0.038–0.524; *p* = 0.003) were significantly associated with differential diagnosis of stage IA EC from BELs. This clinical model achieved an AUC of 0.705 (95% CI 0.638–0.773, sensitivity = 65.1%; specificity = 70.8%).

#### ADC histogram model

After univariate and multivariate regression analysis, ADC_10th_, rMAD, total energy, and skewness were retained to fit the ADC histogram model. ADC-score calculated as the linear combination of these features with the logistic regression model coefficients was as follows:

$${\text{ADC - score}} = \;0.{257} + - {3}.{27}0 \times {\text{ADC}}_{{{1}0{\text{th}}}} { + } - 0.{71}0 \times {\text{ rMAD}} + {1}.0{25} \times {\text{Total}}\_{\text{Energy}} + 0.{6}0{5} \times {\text{Sknewness}}$$

Moreover, when combining clinical parameters and ADC-score, ADC-score was the only significant independent predictor (OR = 2.641, 95% CI 2.045–3.411; p < 0.001). All data from multivariate logistic regression models are summarized in Table 6.

**Table 6:** Results of multivariate logistic regression models for differentiating stage IA EC from BELs

Models	Coefficients	Odds ratio	95% CI	*p* value
**Clinical model**				
Age at diagnosis, years				
≤ 50		Reference		
51–64	1.412	4.106	2.269–7.429	< 0.001
≥ 65	0.574	1.776	0.563–5.604	0.328
Nulliparity				
No		Reference		
Yes	1.433	4.191	1.066–16.472	0.040
Long-term Tamoxifen therapy				
No		Reference		
Yes	− 1.963	0.14	0.038–0.524	0.003
**ADC histogram model**				
ADC_10th_	− 3.270	0.038	0.016–0.092	< 0.001
rMAD	− 0.710	0.492	0.271–0.893	0.020
Total energy	1.025	2.788	1.416–5.490	0.003
Skewness	0.605	1.832	1.155–2.907	0.010
**Combined** **model**				
ADC-score*	0.971	2.641	2.045–3.411	< 0.001
Age at diagnosis, years				
≤ 50		Reference		
51–64	0.711	2.036	0.821–5.046	0.125
≥ 65	1.623	5.068	0.905–28.383	0.065
Nulliparity				
No		Reference		
Yes	0.446	1.562	0.252–9.670	0.631
Long-term Tamoxifen therapy				
No		Reference		
Yes	− 0.846	0.429	0.061–2.996	0.393

rMAD, robust mean absolute deviation

*ADC-score = 0.257 + − 3.270 × ADC_10th_ + − 0.710 × rMAD + 1.025 × Total_Energy + 0.605 × Sknewness. All ADC histogram features were standardized by Z-score normalization

**Fig. 5:**
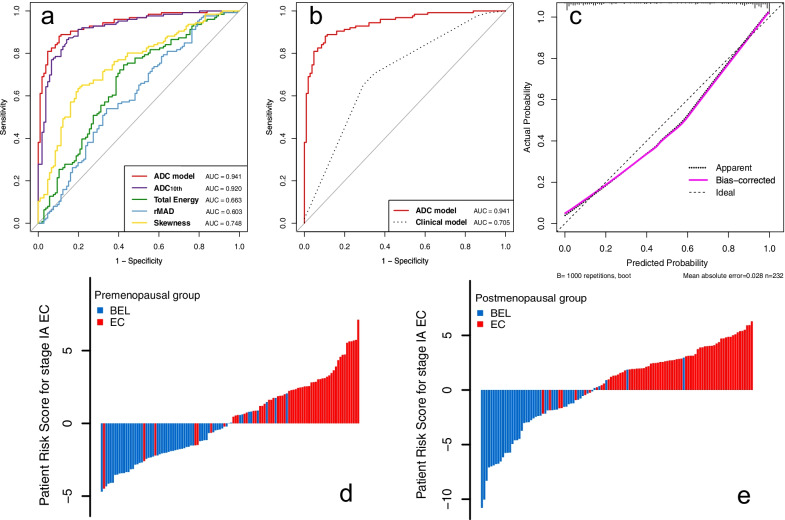
**a** ROCs of the ADC histogram parameters and combined ADC histogram model. **b** ROCs of the ADC histogram model and clinical model. **c** The calibration plot of the ADC histogram model. Patient risk scores output by the ADC histogram model for **d** premenopausal and **e** postmenopausal patients, while red bars show scores for those with stage IA EC. rMAD, robust mean absolute deviation

ROC analysis showed that the ADC histogram model had a significantly higher AUC of 0.941 (95% CI 0.912–0.970, sensitivity = 88.1%; specificity = 89.6%) than the clinical model (*p* < 0.001). Although the AUC of the ADC histogram model was higher than ADC_median_, no significant difference existed (*p* = 0.071). Bias-corrected AUCs generated through an enhanced bootstrap resampling process showed slight reductions for the ADC histogram model, from 0.941 to 0.937. The ROCs of the models are shown in Fig. 5a, b. The calibration curve showed good fitness for the ADC histogram model (Hosmer–Lemeshow test, *p* = 0.504) (Fig. 5c).

Subgroup analysis revealed that the ADC histogram model achieved an AUC of 0.919 (95% CI 0.866–0.973), a sensitivity of 0.881, a specificity of 0.896, and an accuracy of 0.888 in the premenopausal group. The model achieved an even higher AUC of 0.957 (95% CI 0.922–0.991), a sensitivity of 0.886, a specificity of 0.935, and an accuracy of 0.905 in the postmenopausal group. Figure 5d, e shows the performance of the ADC histogram model in the premenopausal and postmenopausal populations intuitively. In addition, the ADC histogram model also performed well in distinguishing BELs from stage IA endometrioid ECs, with an AUC of 0.943 (95% CI 0.913–0.973), a sensitivity of 0.892, a specificity of 0.896, and an accuracy of 0.894.

### Diagnostic efficacy of ADC histogram parameters in characterizing histopathologic features of stage IA EC

Grade 3 stage IA ECs showed significantly lower ADC_min_ and ADC_10th_ values compared to Grade 1/2 tumors (p = 0.022 and 0.047, respectively; Additional file 1: Table S1). ROC analysis showed that ADC_min_ was more effective in comparison to other parameters. Using the Youden index, a threshold value of 0.583 × 10^−3^mm^2^/s for ADC_min_ was identified. This threshold yielded an AUC of 0.641 (95% CI 0.518–0.763), a sensitivity of 40.7%, a specificity of 85.4%, and an accuracy of 75.6% (Additional file 1: Table S2).

The level of the proliferation index Ki-67 was available for 80 EC patients. For the 80 stage IA EC lesions, pathologic evaluation of Ki-67 ranged from 1% to 90% (median, 30%). Spearman’s rank correlation coefficients showed no correlations between ADC histogram parameters and expression of Ki-67 in stage IA EC (all p > 0.05; Additional file 1: Table S3). Also, there were no significant differences between the ADC parameters in low- and high- Ki-67 expression groups (all p > 0.05; Additional file 1: Table S4).

## Discussion

Our study demonstrated that ADC histogram parameters derived from whole-lesion assessment could help distinguish stage IA EC from BELs preoperatively. The ADC_median_ yielded the highest AUC of 0.928 for differentiating BELs from stage IA EC among all the ADC histogram parameters. Furthermore, multivariate analysis showed that ADC-score (ADC_10th_ + skewness + rMAD + total energy) was the only significant independent predictor for stage IA EC when considering the clinical parameters. This ADC histogram model (ADC-score) achieved an AUC of 0.941 and bias-corrected AUC of 0.937 and performed well for premenopausal and postmenopausal patients.

In the present study, the ADC values, including ADC_10th_, ADC_90th_, ADC_min_, ADC_max_, ADC_mean_, ADC_median_, and IQR of stage IA EC, were all significantly lower than those of BELs, which is consistent with previous studies [10, 28, 29]. The denser cellularity in malignant lesions leads to the restriction of water molecular diffusion and corresponding decreased ADC values [12]. Furthermore, most previous studies included EC of different stages with relatively small sample sizes. In this study, we analyzed the capacity of ADC values in the discriminating early-stage EC from BELs. Whereas prior studies mainly focused on the role of standard mean ADC values, we observed that the whole-lesion ADC_median_, ADC_mean_, and ADC_10th_ all showed high classification potential, which could be easily applied in clinical practice.

Several previous studies have suggested that low percentiles of ADC are more helpful in diagnosing and classifying malignancies compared to mean ADC or high percentiles [30–33]. Kierans et al*.* [20] suggested that ADC_10th_ may accurately predict malignant endometrial lesions compared to ADC_mean_. In our study, although ADC_10th_ was not superior to the mean or median ADC in the classification task, it showed a reasonably good differentiating performance and was selected as one of the optimal parameters of the ADC histogram model. A possible explanation is that lower percentiles ADC may better represent aggressive solid components within endometrial malignancies, while the high percentile ADC might be vulnerable to the cystic or necrotic components [34]. In clinical work, such microcystic changes possibly failed to be excluded from the VOI because of the limitation of visual detection. Therefore, it was unsurprising that ADC_10th_ effectively discriminated the two lesions with distinct compactness.

The ADC histogram analysis represents texture-based statistics of the variation and frequency of ADC values within a given tissue. It can assess the deviation of the histogram from a normal distribution as a marker of structural heterogeneity and complexity. Previous studies demonstrated more significant heterogeneity in more aggressive lesions [35–38]. Besides the quantitative ADC values, we found that the histogram parameters, MAD, rMAD, RMS, energy, total energy, entropy, and variance of the stage IA EC were significantly lower. At the same time, skewness, kurtosis, and uniformity were significantly higher than those of BELs. After univariate and multivariate regression analysis, skewness, total energy, and rMAD were included in the final ADC histogram model.

Skewness reflects the asymmetry of the ADC histogram distribution. Positive skewness indicates that most voxels contain ADC values below the mean, and a long tail of the curve leans rightward. Prior studies have demonstrated significantly higher skewness in soft tissue sarcomas than in benign peripheral neurogenic tumors [35], as well as invasive compared to noninvasive intraductal papillary neoplasms of the bile ducts [36]. Therefore, our observation of greater ADC skewness in stage IA EC probably reflects this increased structural heterogeneity within the lesions, with a predominance of lower ADC values indicating the reduction in ADC arising from neoplasia-related cellularity.

Energy refers to the magnitude of voxel values in the image; it is volume-dependent, and larger values imply a higher sum of the squares of these values. The total energy is the value of the energy feature scaled by the voxel volume in cubic mm [24]. Since no significant difference existed in the volume of VOIs between these two groups in the current study, we concluded that stage IA EC had significantly lower energy and total energy than BELs due to lower voxel intensity values within the entire tumor in ADC maps.

In our study, significantly higher rMAD was observed in BELs than in stage IA EC. The rMAD is the mean distance of all intensity values from the mean value calculated on the subset of image array with gray levels in between, or equal to the 10th and 90th percentile, which is robust optimization of the MAD model [24]. A larger MAD indicates a higher contrast between high and low intensity in a tumor. With our effort to exclude the necrotic, cystic, and hemorrhagic areas in the VOIs, we considered that stage IA EC lesions had increased tumor cellularity, resulting in a relatively uniform reduction in ADC values. In contrast, benign lesions, such as endometrial polyps, contain endometrial glands and stroma of focally or diffusely dense fibrous or smooth muscle tissue [6]. Cystic glandular hyperplasia commonly occurs within the polyp. This tissue characterization can cause a more dispersed ADC distribution in the VOIs.

Previous studies demonstrated that endometrial pathologies share common predisposing risk factors, such as age, obesity, diabetes, postmenopausal status, nulliparity, and long-term tamoxifen therapy [39]. Our data suggested that women aged 51–64 were more likely to have EC than BELs than those under 50 or over 65. Similarly, Abid et al*.* [40] found that age was associated with more progressive lesions in peri- and postmenopausal age groups such as EC, yet endometrial polyp was the most common pathology in postmenopausal women. Meanwhile, nulliparity is an established risk factor for endometrial cancer, and each pregnancy provides an additional risk reduction [41]. In contrast, the mechanisms and hormone profiles that underlie alterations in endometrial cancer risk are not fully understood. Prolonged tamoxifen use is associated with an increased incidence of various endometrial lesions, including endometrial polyp, endometrial hyperplasia with or without atypia, EC, and sarcoma [42]. However, compared to EC, most endometrial lesions detected in tamoxifen users are benign, among which endometrial polyps represent the most common endometrial pathology [43, 44]. In the current study, we found that long-term tamoxifen therapy was significantly associated with diagnosing BELs, consistent with prior studies. Nevertheless, the efficacy of the above-mentioned clinical model was moderate, and no clinical parameters survived after multivariate regression analysis. Different from a previous study [20], we proved that the ADC histogram model could predict the presence of malignancy in the postmenopausal and premenopausal groups with larger sample size.

As the tumor progresses, changes in the tumor microenvironment enhance the proliferative ability. ADC values based on water molecular diffusion may reflect the microstructure of relevant tissues in this respect. Recently, numerous studies have analyzed the associations between ADC values and histopathological features like tumor grade and Ki-67 in different tumors [45, 46]. In our research, ADC_min_ and ADC_10th_ values for Grade 3 stage IA EC were significantly lower than those for Grade 1/2 tumors, in line with the results of previous studies [47, 48]. Similar to the differentiation of malignant from benign lesions, the region showing minimum ADC values may reflect the highest cellular area within the tumor, which is more representative of tumor grade or aggressiveness. However, the diagnostic efficiency of ADC_min_ is insufficient, with an AUC of 0.641 and a sensitivity of 40.7%. Also, no significant correlation was found between ADC histogram parameters and the expression of Ki-67 in stage IA EC. Therefore, it is necessary to explore more sensitive indicators to predict tumor aggressiveness of early-stage EC in the future, such as parameters derived from amide proton transfer-weighted imaging, which have shown promising prospects in this regard [26].

This study has a few limitations. First, this was a single-center retrospective study with inherent selection bias. Further external validation in independent data sets with a large number of patients is necessary in the future. Second, we did not investigate the potential value of ADC histogram parameters in classifying BELs as polyps, hyperplasia without atypia, or atypical hyperplasia. Because BELs usually coexist pathologically and can have similar signals on conventional MRI, it is hard to ensure the precision of segmentation. Third, although most endometrial lesions could be contoured on DWI with multimodal MR images as references, there were still obscure boundaries between the lesions and normal endometrium in some BELs, such as endometrial hyperplasia, where we contoured the entire endometrium as VOIs. The bias introduced by inconsistency in VOI drawing was difficult to avoid in clinical practice and minimized by consulting another experienced radiologist in our study. Finally, patients underwent MRI with different MR equipment and protocols. The histogram metrics, rather than the ADC values, can directly reflect the ADC distribution and are hoped to remain reliable despite different MRI systems.

## Conclusions

Our study suggested that whole-lesion ADC histogram analysis can promote preoperative differentiation of stage IA EC from BELs and histopathologic grading of stage IA EC in premenopausal and postmenopausal patients, thereby contributing to clinical treatment planning.

## Supplementary Information

**Additional file 1.** **Table S1.** Comparison of ADC histogram parameters between Grade 1/2 and Grade 3 stage IA EC. **Table S2.** Diagnostic performance of volumetric ADC histogram parameters in differentiating Grade 3 from Grade 1/2 stage IA EC. **Table S3.** Correlation between ADC histogram parameters and expression of Ki-67 in stage IA EC. **Table S4.** Comparison of ADC histogram parameters between stage IA ECs with low- and high- Ki-67 expression.

## Data Availability

All data generated or analysed during this study are included in this published article.

## Abbreviations

ADC: Apparent diffusion coefficient
ADC_10th_: 10th percentile ADC
ADC_90th_: 90th percentile ADC
BELs: Benign endometrial lesions
CI: Confidence interval
DWI: Diffusion-weighted imaging
EC: Endometrial carcinoma
FIGO: International Federation of Gynecology and Obstetrics
ICC: Intraclass correlation coefficients
IQR: Interquartile range
MAD: Mean absolute deviation
MRI: Magnetic resonance imaging
OR: Odds ratio
rMAD: Robust mean absolute deviation
RMS: Root mean squared
ROC: Receiver operating characteristic
VOI: Volume of interest
