# Supplementary material for: Whole-lesion apparent diffusion coefficient (ADC) histogram as a quantitative biomarker to preoperatively differentiate stage IA endometrial carcinoma from benign endometrial lesions
Source: BMC Med Imaging. 2022 Aug 8;22:139. doi: 10.1186/s12880-022-00864-9 (PMC9358891; doi:10.1186/s12880-022-00864-9)
Supplement: Supplementary file 1 — Additional file 1. Table S1. Comparison of ADC histogram parameters between Grade 1/2 and Grade 3 stage IA EC. Table S2. Diagnostic performance of volumetric ADC histogram parameters in differentiating Grade 3 from Grade 1/2 stage IA EC. Table S3. Correlation between ADC histogram parameters and expression of Ki-67 in stage IA EC. Table S4. Comparison of ADC histogram parameters between stage IA ECs with low- and high- Ki-67 expression. [file 12880_2022_864_MOESM1_ESM.docx]

**Supplementary Material**

| **Table S1.** Comparison of ADC histogram parameters between Grade 1/2 and Grade 3 stage IA EC | | | |
| --- | --- | --- | --- |
| Histogram parameters | Grade 1/2^#^ | Grade 3^#^ | *p* value |
|  | (n=98) | (n=28) |  |
| **Volume** (×10^3^mm^3^) | 4.741 (2.250, 9.789) | 7.800 (3.164, 14.602) | 0.114 |
| **ADC_10th_** (×10^-3^mm^2^/s) | 0.819 (0.749, 0.909) | 0.771 (0.679, 0.854) | **0.047** |
| **ADC_90th_** (×10^-3^mm^2^/s) | 1.191 (1.102,1.305) | 1.128 (1.021, 1.354) | 0.492 |
| **ADC_min_** (×10^-3^mm^2^/s) | 0.709±0.138 | 0.636±0.170 | **0.022** |
| **ADC_max_** (×10^-3^mm^2^/s) | 1.506 (1.324, 1.685) | 1.537(1.337, 1.781) | 0.555 |
| **ADC_mean_** (×10^-3^mm^2^/s) | 0.989 (0.908, 1.090) | 0.961 (0.843, 1.052) | 0.160 |
| **ADC_median_** (×10^-3^mm^2^/s) | 0.970 (0.891, 1.073) | 0.950 (0.816, 1.032) | 0.205 |
| **IQR** (×10^-3^mm^2^/s) | 0.186 (0.152, 0.235) | 0.195 (0.181, 0.259) | 0.174 |
| **Range** (×10^-3^mm^2^/s) | 0.800 (0.621, 1.004) | 0.875 (0.736, 1.189) | 0.093 |
| **MAD** (×10^-3^) | 0.109 (0.097, 0.140) | 0.122 (0.106, 0.157) | 0.153 |
| **rMAD** (×10^-3^) | 0.078 (0.063, 0.097) | 0.083 (0.075, 0.110) | 0.148 |
| **RMS** (×10^-3^) | 1.003 (0.916, 1.106) | 0.979 (0.864, 1.070) | 0.183 |
| **Energy** (×10^-3^) | 0.331 (0.155, 0.694) | 0.383 (0.264, 1.160) | 0.179 |
| **Total Energy** (×10^-3^) | 4.900 (2.151, 9.887) | 6.054 (3.442, 16.877) | 0.206 |
| **Entropy** (×10^-6^) | 4.016 ± 0.445 | 4.126 ± 0.350 | 0.238 |
| **Skewness** (×10^-6^) | 0.669 (0.284, 1.059) | 0.918 (0.349, 1.192) | 0.334 |
| **Kurtosis** (×10^-6^) | 3.325 (2.700, 4.734) | 3.952 (2.827, 4.436) | 0.517 |
| **Variance** (×10^-6^) | 0.021(0.015, 0.034) | 0.023 (0.015, 0.042) | 0.258 |
| **Uniformity** (×10^-6^) | 0.074(0.060, 0.090) | 0.071 (0.056, 0.081) | 0.299 |
| ^#^Data are mean ± standard deviation (normal distribution) or median and interquartile range (non-normal distribution), evaluated using the Kolmogorov-Smirnov test | | | |
| IQR, Interquartile Range; MAD, Mean Absolute Deviation; rMAD, Robust Mean Absolute Deviation; ICC, intraclass correlation coefficients | | | |

| **Table S2.** Diagnostic performance of volumetric ADC histogram parameters in differentiating Grade 3 from Grade 1/2 stage IA EC | | | | | |
| --- | --- | --- | --- | --- | --- |
| Histogram parameters | AUC (95%CI) | Sensitivity (%) | Specificity (%) | Accuracy (%) | Cut-off value |
| **ADC_10th_** (×10^-3^mm^2^/s) | 0.625 (0.505-0.745)  0.641 (0.518-0.763) | 48.1 | 77.1 | 70.7 | 0.744 |
| **ADC_min_** (×10^-3^mm^2^/s) |  | 40.7 | 85.4 | 75.6 | 0.583 |

| **Table S3**. Correlation between ADC histogram parameters and expression of Ki-67 in stage IA EC | | |
| --- | --- | --- |
| Parameters | Correlation coefficients | *p* value |
| **Volume** | -0.067 | 0.557 |
| **ADC_10th_** | -0.079 | 0.488 |
| **ADC_90th_** | 0.018 | 0.871 |
| **ADC_min_** | -0.007 | 0.952 |
| **ADC_max_** | 0.131 | 0.245 |
| **ADC_mean_** | -0.007 | 0.952 |
| **ADC_median_** | -0.017 | 0.881 |
| **IQR** | 0.200 | 0.075 |
| **Range** | 0.212 | 0.058 |
| **MAD** | 0.200 | 0.076 |
| **rMAD** | 0.207 | 0.066 |
| **RMS** | 0.005 | 0.963 |
| **Energy** | -0.013 | 0.907 |
| **Total Energy** | -0.053 | 0.640 |
| **Entropy** | 0.130 | 0.249 |
| **Skewness** | 0.020 | 0.860 |
| **Kurtosis** | 0.055 | 0.630 |
| **Variance** | 0.203 | 0.071 |
| **Uniformity** | -0.138 | 0.221 |

| **Table S4.** Comparison of ADC histogram parameters between stage IA ECs with low- and high- Ki-67 expression | | | |
| --- | --- | --- | --- |
| Histogram features | Low expression^*^ | High expression^*^ | *p* value |
|  | (n=29) | (n=51) |  |
| **Volume** (×10^3^mm^3^) | 5.833 (3.596, 9.927) | 7.800 (3.164, 14.602) | 0.837 |
| **ADC_10th_** (×10^-3^mm^2^/s) | 0.814 (0.751, 0.894) | 0.799 (0.696, 0.872) | 0.522 |
| **ADC_90th_** (×10^-3^mm^2^/s) | 1.170 (1.112,1.268) | 1.187 (1.024, 1.323) | 0.674 |
| **ADC_min_** (×10^-3^mm^2^/s) | 0.693±0.164 | 0.683±0.145 | 0.797 |
| **ADC_max_** (×10^-3^mm^2^/s) | 1.434 (1.329, 1.567) | 1.537(1.337, 1.765) | 0.108 |
| **ADC_mean_** (×10^-3^mm^2^/s) | 0.988 (0.915, 1.045) | 0.991 (0.869, 1.083) | 0.996 |
| **ADC_median_** (×10^-3^mm^2^/s) | 0.982 (0.885, 1.026) | 0.967 (0.844, 1.060) | 0.826 |
| **IQR** (×10^-3^mm^2^/s) | 0.170 (0.128, 0.230) | 0.195 (0.158, 0.248) | 0.084 |
| **Range** (×10^-3^mm^2^/s) | 0.596 (0.491, 0.690) | 0.659 (0.478, 0.824) | 0.053 |
| **MAD** (×10^-3^) | 0.106 (0.083, 0.133) | 0.111 (0.099, 0.149) | 0.071 |
| **rMAD** (×10^-3^) | 0.071 (0.055, 0.094) | 0.080 (0.067, 0.107) | 0.057 |
| **RMS** (×10^-3^) | 0.995 (0.938, 1.062) | 1.004 (0.890, 1.100) | 0.877 |
| **Energy** (×10^-3^) | 0.397 (0.226, 0.557) | 0.361 (0.155, 0.977) | 0.745 |
| **Total Energy** (×10^-3^) | 5.819 (3.312, 9.071) | 5.678 (2.438, 12.147) | 0.980 |
| **Entropy** (×10^-6^) | 3.934 ± 0.409 | 4.064 ± 0.458 | 0.196 |
| **Skewness** (×10^-6^) | 0.665 (0.215, 1.007) | 0.769 (0.320, 1.171) | 0.512 |
| **Kurtosis** (×10^-6^) | 3.289 (2.838, 4.252) | 3.467 (2.743, 4.812) | 0.799 |
| **Variance** (×10^-6^) | 0.017(0.011, 0.026) | 0.022 (0.016, 0.037) | 0.065 |
| **Uniformity** (×10^-6^) | 0.075(0.061, 0.098) | 0.073 (0.059, 0.086) | 0.252 |
| ^*^Data are mean ± standard deviation (normal distribution) or median and interquartile range (non-normal distribution), evaluated using the Kolmogorov-Smirnov test | | | |
| IQR, Interquartile Range; MAD, Mean Absolute Deviation; rMAD, Robust Mean Absolute Deviation; ICC, intraclass correlation coefficients | | | |
